# Anonymous Letters from Cortland Co., N. Y.

**Published:** 1847-02

**Authors:** 


*Anonymous Letters from Cortland Co., N. Y.*—A while since, anonymous letters, of an extremely provoking character, came repeatedly through the mail from the western part of New-York State. They were the offspring of a malevolent disposition, and the outpourings of some one who was provoked at the rising eminence of a gentleman whose name occasionally appeared in our pages. It was the writer's object to impress us with the idea that he was a member of the Cortland Medical Society. Ever alive to the honor of the Association, whose good name is above rubies, unsolicited on our part, an investigation was instituted by a vigilant committee of the Society, to ascertain whether it was possible any member could have so demeaned himself as to have written in such a reprehensible manner, as cowardly as it was ungentlemanly. The result of the investigation has proved that no gentleman belonging to the Cortland County Medical Society was ever guilty of the disreputable act. It was the work of an assassin—a villain who would cut a neighbor's throat as quickly as he would ruin his reputation, and who assumed to belong to a body that was never yet disgraced by one of his odious propensities.—*Boston Med. and Surg. Jour.*

